# Calcifying aponeurotic fibroma of the thigh: A differential diagnosis for infant knee contracture and leg length inequality (a case report)

**DOI:** 10.1016/j.ijscr.2020.03.039

**Published:** 2020-04-01

**Authors:** Liuzhe Zhang, Hiroshi Kobayashi, Masachika Ikegami, Takahiro Ohki, Yusuke Shinoda, Sakae Tanaka, Hirotaka Kawano

**Affiliations:** a Department of Orthopaedic Surgery, Faculty of Medicine, The University of Tokyo, 7-3-1 Hongo, Bunkyo-ku, Tokyo 113-8655, Japan; b Department of Orthopaedic Surgery, Faculty of Medicine, Teikyo University, 2-11-1, Kaga, Itabashi-ku, Tokyo 173-8606, Japan

**Keywords:** CAF, calcifying aponeurotic fibroma, MRI, magnetic resonance imaging, Calcifying aponeurotic fibroma, Infant knee contracture, Leg length inequality, Case report

## Abstract

**Introduction:**

Calcifying aponeurotic fibroma is an uncommon type of tumor that primarily occurs in the distal extremities of young children. It usually appears as a slow-growing and asymptomatic soft-tissue mass and rarely causes complications, such as joint contractures.

**Presentation of case:**

We present a case of calcifying aponeurotic fibroma that caused knee contracture and leg length inequality, in which the affected leg was longer than the other. On the patient’s first visit to our hospital at 8 years of age, a solid nodule was palpable at the medial distal area of her right thigh. Magnetic resonance imaging showed a T1WI-low, fs-T2WI-slightly high 60-mm lesion on the vastus intermedius muscle with enhancement caused by a gadolinium agent. Tumor resection surgery, which was preceded by an incisional biopsy that resulted in calcifying aponeurotic fibroma, improved the knee’s range of motion (extension-flexion improved from 0–80° to 0–120°). For the remaining leg length inequality of 35 mm, epiphyseal arrest surgery is planned.

**Discussion and conclusion:**

Very infrequently, calcifying aponeurotic fibroma can cause infant knee contracture and leg length inequality. These symptoms were seemingly caused, respectively, by fascia adhesion and by increased blood flow around the metaphysis. Timely diagnosis and appropriate surgical intervention may improve functional prognosis in affected patients.

## Highlights

• Calcifying aponeurotic fibroma is a rare tumor primarily in the distal extremities.
• Very rarely, this tumor can cause infant knee contracture and leg length inequality.
• The mechanism may be fascia adhesion and increased blood flow around the metaphysis.
• Timely diagnosis and appropriate surgical intervention may improve functional prognosis.

## 1 Introduction

In 1953, Keasbey described a slow-growing, benign, painless tumor that occurred in the fingers and palms of juvenile and adolescent patients and named it juvenile aponeurotic fibroma [1]. Since then, it has become known that it can affect a wider age range, so the term calcifying aponeurotic fibroma (CAF) has become preferred. Additionally, this lesion is now known to affect other parts of the body; it chiefly affects the distal extremities, but it can also affect other sites, including the elbow, scalp, gluteal region, and others [2,3]. Most patients present with a slowly growing painless mass of several months’ or years’ duration, which usually cause no discomfort or little functional impairment [4].

In this report, we present a case in which CAF of the thigh might have caused knee contracture and leg length inequality, in which the affected leg was longer. This case report is in line with the SCARE criteria [5].

## 2 Presentation of case

An 8-year old Japanese girl was referred to our university hospital with history of right knee contracture, leg length inequality and a right thigh tumor. When the patient was a few months old, her parents noticed her toddling poorly with difficulty bending the right knee. By 2 years of age, the contracture of the right knee and the inequality in leg length (the right leg was longer) had become more apparent, so the shoes with sole lifts were prescribed by the primary physician. The contracture and the leg length inequality were resistant to conservative treatment, and by the time the patient was 8 years old, she and her parents had noticed a tumor palpable at her medial thigh. The patient was, therefore, referred to our hospital for further evaluation.

On physical examination at our clinic, the affected leg was 35 mm longer than the opposite leg, and the range of motion of the right knee was limited (extension-flexion range was 0–80° on the right and 0–140° on the left) (Fig. 2a). A 4 × 4-cm elastic-hard nodule was palpable at the distal frontal to medial thigh. The patient complained of mild tenderness on palpation.

**Fig. 1:**
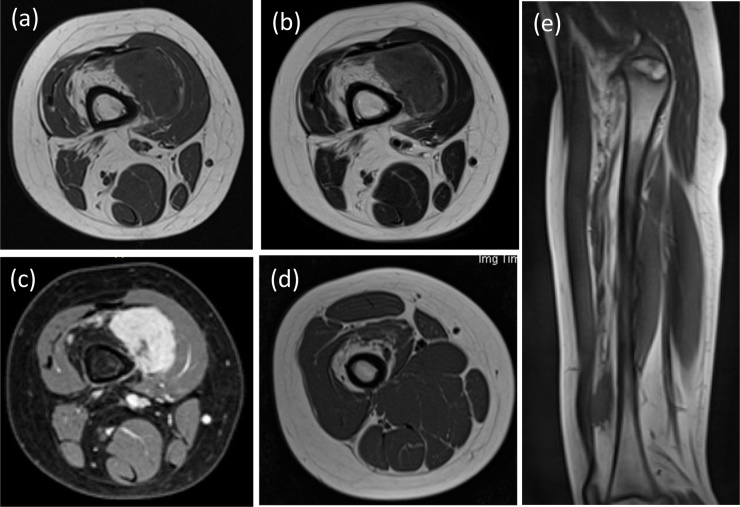
Pre-operative magnetic resonance image of the right femur. The lesion was located beneath the vastus medialis muscle, and the vastus intermedius muscle showed fatty degeneration. Axial view of the right distal femur on (a) T1-weighted imaging, (b) T2-weighted imaging, and (c) gadolinium-enhanced, T1-weighted, fat-suppressed imaging. (d) Axial view of the right proximal femur and (e) sagittal view of the right femur on T1-weighted imaging show that fatty degeneration stretched the full length of the vastus intermedius muscle.

Magnetic resonance imaging (MRI) revealed a mass with low-signal intensity compared to that of the muscle on T1-weighted images and slightly high signal intensity on T2-weighted images; the mass was located under the vastus medialis muscle and merged into the vastus intermedius muscle (Fig. 1a, b). The lesion showed intense, heterogeneous gadolinium enhancement on T1-weighted fat-suppressed images (Fig. 1c). A considerable part of the vastus intermedius muscle appeared to have turned into fat tissue with some fibrous elements (Fig. 1d, e). The incisional biopsy revealed histopathological findings consistent with those of CAF.

**Fig. 2:**
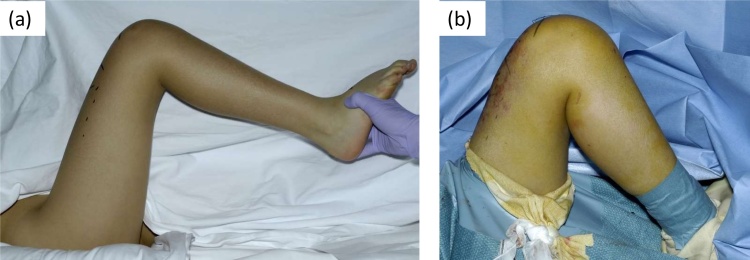
Limited range of motion in the right knee was relieved by tumor resection. (a) Under general anesthesia, the affected knee had some flexion contracture preoperatively, with a range of motion of 0–90°. (b) Postoperatively, the knee contracture had improved, with a range of motion of 0–130°.

**Fig. 3:**
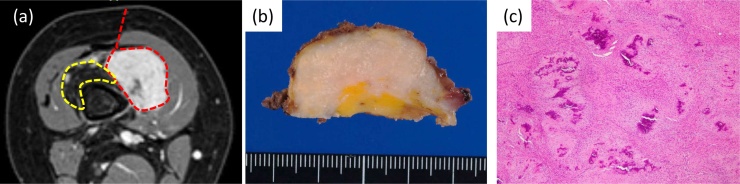
Surgical margin, macroscopic findings, and tumor histology. (a) The lesion was marginally resected (red dotted line) along the distal intermediate vastus muscle and its fascia (yellow dotted line). (b) Grossly, the specimen was oval and as large as 3 cm at its greatest diameter. On the resected specimen, small white flecks were observed, suggesting calcification. (c) Histologically, the lesion had multiple calcifications surrounded by round cells arranged in short arrays. The stroma was largely hyalinized.

Since the tumor seemed to be the culprit of the symptom, which was resistant to physical therapy, we decided to perform tumor resection surgery. At surgery, the tumor was surrounded by a significantly fatty-degenerated vastus intermedius muscle, conglutinated to the fascia of the vastus intermedius muscle, and extended very close to both the vastus medialis and lateralis muscles. We primarily resected the lesion on the vastus intermedius muscle, but the range of motion remained unchanged compared to that pre-surgically due to the tethering of the remaining fascia of the vastus intermedius muscle, while the rectus femoris muscle showed little tightness with the knee flexed. Since the primary goal of the surgery was to improve the range of motion of the knee, an additional transverse cutting of the fascia of the vastus intermedius muscle successfully relieved the tension and improved the knee flexion to 120° (Fig. 2b). We resected the distal vastus intermedius muscle and its fascia, but laterally only at the transient zone to the vastus medialis and lateralis muscles in order to preserve the extension function, running the risk of local recurrence (as agreed with the patient’s parents) (Fig. 3).

**Fig. 4:**
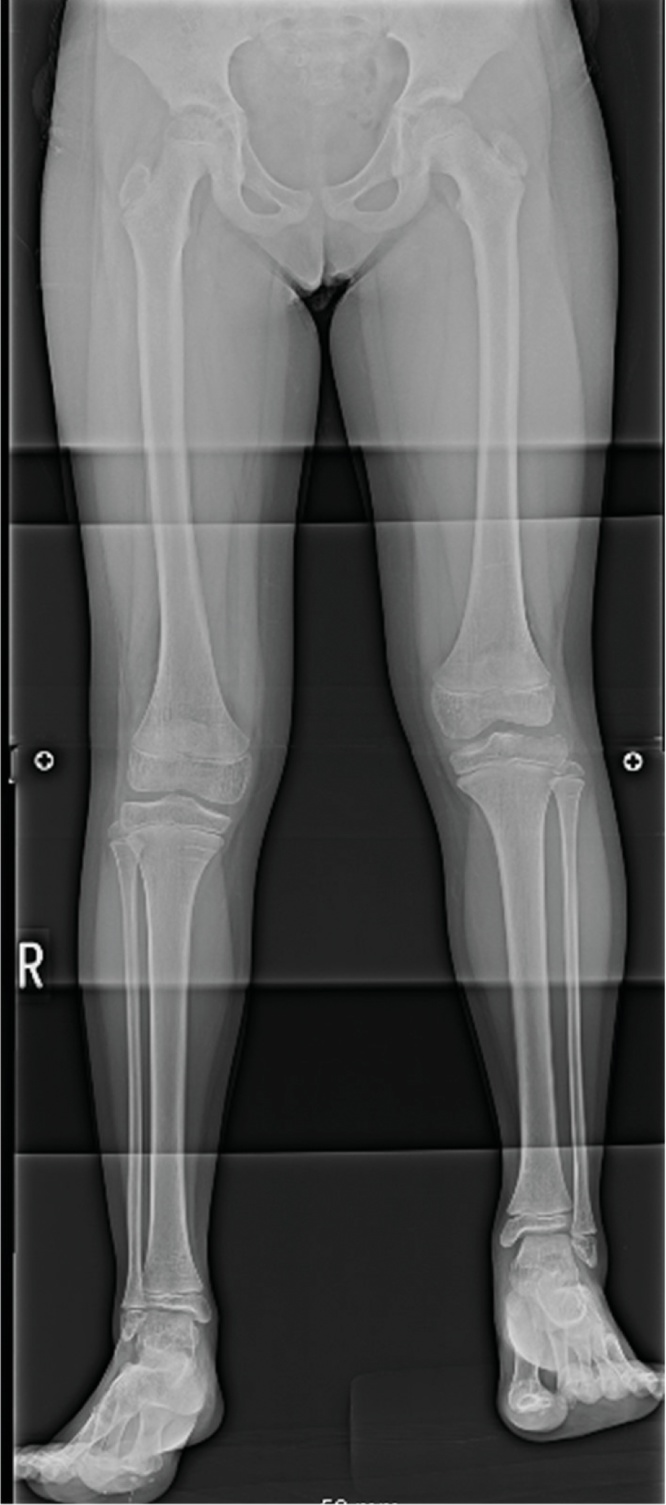
Plain radiographs of both lower extremities. Plain radiography showed that limb inequality remained even 1 year after tumor resection.

As of the 12th month after surgery, the knee extension-flexion range has been maintained at 0–110°, and the leg length discrepancy has not shown an apparent increase from 35 mm (Fig. 4). MRI and echography suggested a residual lesion in the distal medial vastus, with no apparent growth in size over the 12-month period. Now, the patient is freely able to run, ride a bicycle, and jump rope. Epiphyseal arrest surgery is planned to remove the need for the patient to wear the shoes with sole lifts.

## 3 Discussion

We described the case of a girl with a CAF located on the deep layer of the anterior thigh that caused knee contracture and leg length inequality. This case report has three major points.

The first point is that CAF, a usually symptom-free tumor, caused a refractory knee contracture. CAF is reported to be a slowly growing, benign, painless tumor that generally causes neither discomfort nor limitations in movement, with a few reports of complaint of mild tenderness [6]. In this case, however, the lesion caused knee contracture, and so this is the first detailed report of such a case, to our best knowledge based on a literature research. Since most of the past reports on CAF around the knee have not mentioned contracture, there might be some additional factor that caused the contracture in this case. Considering that the simple tumor excision did not immediately improve the flexion range but that the additional incision of fascia did, the hardening and shortening of the fascia of the vastus intermedius muscle might be the key culprit. This hardening and shortening were probably due first to the invasion of the tumor and second to the years of disuse since birth caused by the discomfort or mild pain on motion. This mechanism provides an interesting analogy to that of quadriceps muscle contracture, an important differential diagnosis of knee contracture of children. Its histological findings are an excess of fibrous tissue and infiltration of fatty tissue, and its treatment is the incision of affected muscles and fascia, which is quite similar to our approach in this case [7].

The second major point is the conservative surgical strategy. CAF’s microscopic infiltrative nature and predilection for local recurrence are well reported, so complete excision generally requires a wide excision margin. However, CAF is considered to have an initial growth phase of rapid infiltration and a late growth phase of prominent calcification, and conservative excision is more reasonable for the tumor in the late phase to maintain the function of the extremity [8]. Additionally, malignant conversion of this disease is very rare [9,10]. In this case, complete excision might have caused more damage to the vastus medialis and rectus femoris muscles, subsequently compromising knee extension function. Therefore, we only excised the lesion in the vastus intermedius muscle. The lesion was assumed not to grow vigorously going forward, since its long history and the prominent calcifications suggested it was in its late growth phase.

The third major point is the mechanism causing the leg length inequality. Based on our research, this is the first report on leg length inequality related to CAF around the knee. The inequality was mainly derived from the femoral bone (femoral bone length: 371/340 mm, tibia bone length: 273/266). No length inequality of the upper extremities nor soft-tissue hypertrophy of the affected leg were observed, which made a diagnosis of hemi-hypertrophy less likely; no skin or vascular abnormalities suggesting systemic syndromes such as Klippel-Weber syndrome were noted. Additionally, the shorter, unaffected leg showed no history or signs of previous fracture, previous infection, multiple enchondroma, hip joint pathology, or neuromuscular disorder [[11], [12], [13]]. Regarding the fact that leg length inequality is rarely associated with either idiopathic or intramuscular-injection-induced quadriceps muscle contracture, it is assumed to be caused by the tumor itself, not secondary to the contracture. Our hypothesis is that the lesion had infiltrated as deep as the inside of the vastus intermedius and had come close to the distal femoral growth plate, potentially stimulating it in some way. Hemangiomas are reported to cause the overgrowth of long bones when they come close to a growth plate, which etiology is assumed to be the stimulation to the growth plate by increased tumoral blood flow [14,15]. CAFs may be rich in blood flow, especially during the rapidly growing initial phase [7], but no case of CAF with long bone overgrowth has ever been reported. More accumulation of cases and careful clinical observation are required.

## 4 Conclusion

We present a case of CAF that was most likely present at birth and that caused knee contracture and leg length inequality. Very infrequently, CAF can cause infant knee contracture and leg length inequality. Timely diagnosis and appropriate surgical intervention may improve functional prognosis.

## Declaration of Competing Interest

None.

## Sources of funding

None.

## Ethical approval

The ethical approval was obtained from the ethics committee in the University of Tokyo Hospital (reference number: 11019).

## Consent

Written informed consent was obtained from the parents of the patient for publication of this case report and accompanying images.

A copy of the written consent is available for review by the Editor-in-Chief of this journal on request.

## Author contribution

LZ and HK: conceptualization, data curation, and writing – original draft; ST, HK, and YS: conceptualization, writing – review and revision, and supervision; TO and MI: writing – review and revision. All authors read and approved the final manuscript.

## Registration of research studies

Not applicable.

## Guarantor

The guarantor for this study is Hiroshi Kobayashi, the corresponding author of this paper.

## Provenance and peer review

Not commissioned, externally peer-reviewed.
